# Glucagon-like Peptide-1 Receptor Agonists—A Potential New Medication for Pediatric Metabolic-Dysfunction-Associated Steatotic Liver Disease (MASLD)

**DOI:** 10.3390/children11030275

**Published:** 2024-02-23

**Authors:** Erika Choi, Ana Ramirez Tovar, Zhulin He, Dellys M. Soler Rodriguez, Miriam B. Vos, Shruthi Arora, Doris Fadoju

**Affiliations:** 1 Division of Endocrinology, Department of Pediatrics, Emory University, Atlanta, GA 30322, USA; erika.choi@emory.edu (E.C.); doris.o.fadoju@emory.edu (D.F.); 2 Children’s Healthcare of Atlanta, Atlanta, GA 30322, USA; dsolerr@emory.edu (D.M.S.R.); mvos@emory.edu (M.B.V.); 3 Department of Pediatrics, Division of Gastroenterology, Hepatology, and Nutrition, Emory University, Atlanta, GA 30322, USA; ana.maria.ramirez.tovar@emory.edu; 4 Pediatric Biostatistics Core, Emory University, Atlanta, GA 30322, USA; zhulin.he@emory.edu

**Keywords:** ALT, obesity, liver steatosis, MASLD, GLP-1

## Abstract

Metabolic-dysfunction-associated steatotic liver disease (MASLD) is the most common liver disease in children in the US and, if untreated, may progress to end-stage liver disease. Glucagon-like peptide-1 receptor agonists (GLP-1 RAs) have shown improvement in MASLD markers in adults with type 2 diabetes mellitus (T2DM). Currently, there is a lack of medications available for the treatment of pediatric MASLD. We aimed to provide preliminary data on the effects of GLP-1 RAs on markers of MASLD in a retrospective study, in an effort to bridge this gap in the pharmacotherapies available. Nine patients from a T2DM clinic who met the following inclusion criteria were included in this study: patients diagnosed with pre-diabetes or T2DM, prescribed a GLP-1 RA in the prior 12 months, and having alanine aminotransferase (ALT) elevated to twice the upper limit of the normal range, indicating evidence of MASLD. The average change between baseline and the first measurement after starting a GLP-1 RA was calculated for ALT, hemoglobin A1c, and BMI. ALT decreased by an average of 98 points. A1c decreased by an average of 2.2 points. BMI decreased by an average of 2.4 points. There was greater reduction in ALT and A1c compared to BMI, suggesting that improvement in MASLD may be independent of weight loss. This is a preliminary study that shows potential, and prospective studies are needed to evaluate the effects of GLP-1 RAs in the management of pediatric MASLD.

## 1. Introduction

Non-alcoholic fatty liver disease (NAFLD) is a chronic disease characterized by the accumulation of lipids in the liver. The disease ranges from steatosis without inflammation to hepatic steatosis with or without features of fibrosis [1]. The name NAFLD recently changed to metabolic-dysfunction-associated steatotic liver disease (MASLD) and is defined as hepatic steatosis accompanied by any cardiometabolic risk factor. For this study, the term MASLD will be used [2]. MASLD has become the most common liver disease among children in the US, rising alongside an increase in obesity and type 2 diabetes (T2DM) [1]. MASLD can progress to advanced fibrosis, cirrhosis, or hepatic carcinoma if untreated; hence, MASLD is one of the leading causes for liver transplantation in adults [3]. In the United States, the prevalence of steatosis in adolescents 12 to 18 years old has been reported to be around 24–26% on a cohort of patients participating in the National Health and Nutrition Examination Survey (NHANES) [4,5]. In 2022, it was reported that among these patients, 87.7% fulfilled the criteria of MASLD [5]. Among children with obesity, the prevalence of MASLD ranges from 29% to 38% [1]. Its presence is strongly associated with features of metabolic syndrome, increasing risk for insulin resistance, T2DM, and cardiovascular disease [4,5,6], hence the update to its name. A recent study showed the prevalence of T2DM to be 16.8% in a pediatric population with known NAFLD, who now qualify as patients with MASLD. In addition, patients who had more severe disease on liver histology had a higher risk of developing T2DM [7].

MASLD has a strong genetic component but interacts with environmental exposures that drive insulin resistance and obesity [8]. An increase in both systemic and hepatic insulin resistance leads to increased lipolysis, resulting in increased free fatty acid levels. This process stimulates pro-inflammatory cytokine release from the liver and adipose tissue, which further exacerbate insulin resistance, dysfunctional adipose tissue, hepatic inflammation, and liver fibrosis contributing to the development of MASLD and T2DM [9]. In adult populations with hepatic steatosis, insulin resistance and dyslipidemia are more severe in those with T2DM than those without T2DM [10].

Currently, there is no effective pharmacotherapy available for the management of MASLD in the pediatric population. With lifestyle modification alone, achieving the weight loss required to resolve or reverse obesity-associated comorbidities is difficult and requires a holistic approach. However, studies of glucagon-like peptide-1 (GLP-1), an incretin hormone acting on pancreatic beta cells to potentiate insulin secretion and suppress glucagon secretion [11], have shown evidence to suggest additional anti-inflammatory effects resulting in increased insulin sensitivity in hepatic and adipose tissues and decreased hepatic lipogenesis [12]. In adults with T2DM, GLP-1 receptor agonists (GLP-1 RAs) have been shown to have several positive effects through improving glycemic control, reducing appetite, and inducing weight loss through lowering subcutaneous and visceral fat levels [13], as well as reducing risk for cardiovascular events through anti-inflammatory mechanisms [12,14,15]. A phase 2 trial in adults tested a GLP-1 RA for MASLD and a phase 3 trial is underway; however, few data are available regarding the response of MASLD in children to GLP-1 RAs [16].

Our main objective in this study is to evaluate the change in ALT following the use of a GLP1-RA in children with both elevated ALT and T2DM. We aim to provide preliminary data for GL1-RA use as a therapeutic agent for pediatric MASLD.

## 2. Methods

This is a retrospective case series performed with patient data available on the electronic health records (EHRs) of the Children’s Healthcare of Atlanta (CHOA) Type 2 Diabetes Clinic. The protocol was written by the authors, and approval was obtained from Emory University and CHOA IRBs to gather the patient data. Informed consent was waived.

**Figure 1:**
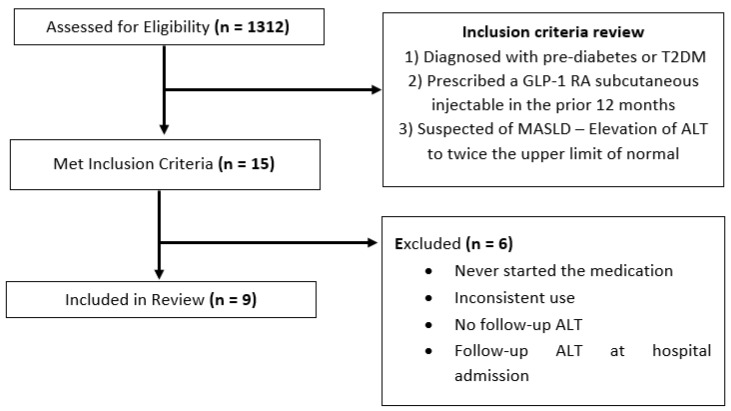
Patient identification flowchart.

A search was conducted using CHOA’s EHRs, Epic. The inclusion criteria were as follows: (1) diagnosed with pre-diabetes or type 2 diabetes mellitus, (2) prescribed and initiated on a GLP-1 RA subcutaneous injectable in the preceding 12 months, and (3) suspected or confirmed MASLD as indicated by alanine aminotransferase (ALT) elevated to more than twice the upper limit of the normal range (>50 U/L for males, >44 U/L for females) in the prior six months in the absence of other liver diseases or a clinician-provided diagnosis of MASLD. A study by Schwimmer et al. found this ALT cut off to have a sensitivity of 88% and specificity of 26% in the diagnosis of MASLD in obese children older than 10 years old [17]. Individual insurance coverage determined the type of GLP-1 RA used for each patient. All currently approved GLP1-RAs for children were included. Clinic charts were reviewed to determine compliance to the medication. ALT, hemoglobin A1c (HbA1c or A1c), and body mass index (BMI) values were obtained. Five patients were excluded for never having started the medication, using the medication inconsistently, or not having a follow-up ALT. One patient was excluded, as follow-up ALT measurements were obtained during a hospital admission (see Figure 1). One 20-year-old patient was included because patients can be seen up to the age of 21 at CHOA.

Baseline and prior-to-treatment status will be used interchangeably to signify the measurements performed before the start of the medication. The primary outcome was percent change and change in absolute value of ALT in relation to starting the GLP-1 RA. Secondary outcomes included percent change and change in absolute value for BMI and change in absolute value for A1c. The baseline ALT before starting medication was an average of ALT values in the 6 months leading to the start of medication. Baseline BMI and HbA1c used was the last value recorded before starting the medication. Average change, median, and range between time points in ALT, BMI, and A1c measurements were calculated and presented with interquartile ranges (IQs).

## 3. Results

A total of 1312 pediatric patients from the T2DM clinic were identified. Charts were initially identified by screening for those that had been prescribed a GLP-1 RA in the specified time period (within the past 12 months). Fifteen charts were identified and screened for full inclusion. Nine patients met the full inclusion and exclusion criteria (see Figure 1). Patient demographics were as follows: six patients were female, and three patients were male. Six patients identified as Hispanic/Latino, two identified as Black, and one identified as White. Participant’s ages ranged from 14 to 20 years.

**Figure 2:**
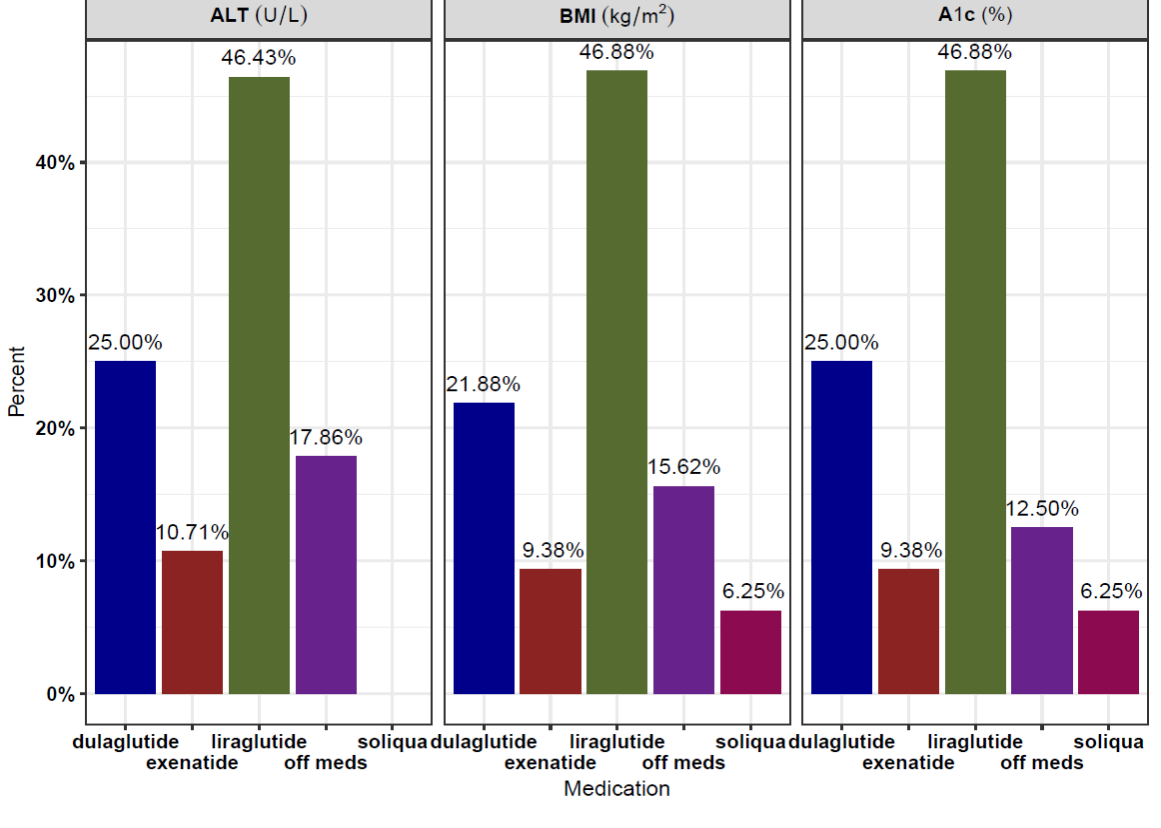
Medication usage, expressed as proportions, for A1c, ALT, and BMI, respectively, across baseline and all follow-up visits.

The GLP-1 RAs used included dulaglutide (3 patients), liraglutide (5 patients), exenatide (1 patient), and a combination drug of glargine and lixisenatide (brand name Soliqua, 1 patient). Figure 2 summarizes the proportion of medication usage for A1c, ALT, and BMI, respectively, at baseline and all follow-up visits. All patients were on optimal doses of GLP-1 RAs (dulaglutide 1.5 mg weekly, exenatide 2 mg weekly, or liraglutide 1.8 mg daily) at the time of measurement. All nine patients had at least one follow-up (or subsequent) ALT, HbA1c, and BMI recorded following initiation of the GLP-1 RA. Six patients had a second follow-up ALT, eight had a second follow-up BMI, and seven had a second follow-up A1c. There was a notable decrease in patients with more than two follow-up values for all three parameters.

Six out of nine patients were on metformin prior to initiation of the GLP-1 RA, and three were on both metformin and insulin. Two patients were started on metformin with the GLP-1RA concomitantly or in the prior 2 months. One patient was started on metformin and insulin in the month prior to the start of the GLP-1 RA. Two patients reported GI side effects including nausea and diarrhea.

**Figure 3:**
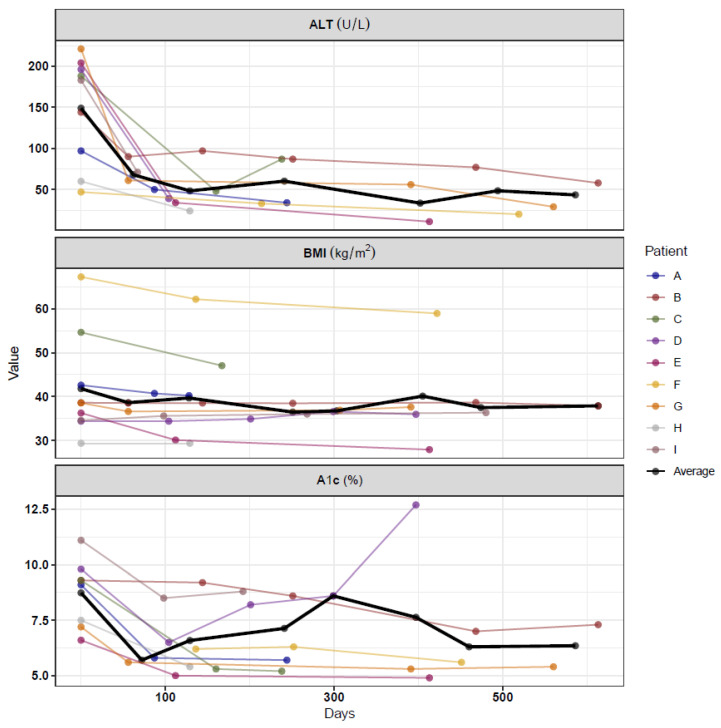
Absolute change per metabolic marker.

Overall, patients were on a GLP-1 RA for an average of 321 ± 210 days from the day of initiation to the day of the last ALT measurement. Six patients were still taking the medication at the time of data collection. Overall, the mean decrease in ALT was 107 points or down 69% from baseline, BMI decreased by 2.76 points or down 5.5% from baseline, and A1c decreased by 2.0 points. The most dramatic decrease in ALT was seen in the first 180 days after starting treatment with the GLP-1 RA (Figure 3). The change in BMI was not as evident. Figure 3 shows the measures of A1c, ALT, and BMI for each patient at baseline and all follow-up visits.

The first clinical follow-up time-point at which labs were obtained varied for each patient. However, the mean time frame was 109 ± 52 days for the first ALT measurement. During the initial follow-up assessment, the median ALT level was 48 U/L (34, 61), decreased from 183 (97, 196), corresponding to a percentage decrease of 61.2% (48.5%, 74.5%) from baseline. The median BMI was 36.6 (34.4, 40.7), representing a percentage decrease of 4.4% (0.03%, 7.6%) from baseline. The median A1c was 5.8% (5.4, 6.5), indicating a percent decrease of 28.0% (23.4%, 34.7%) from baseline.

The average time between baseline and the final follow-up for ALT, A1C, and BMI was 321 ± 210, 360 ± 168, and 349 ± 170 days, respectively (Figure 3). At this final clinical follow-up time point, the median ALT was 34 U/L (24, 58), corresponding to a percentage decrease of 61.2% (59.7%, 80.1%) from baseline. The median BMI was 37.6 kg/m^2^ (36.0, 40.2), representing a percent decrease of 2.4% (0.0%, 12.3%) from baseline. The median A1c was 5.6% (5.4, 6.8), indicating a percent decrease of 25.8% (21.5%, 37.4%) from baseline.

## 4. Discussion

In our cohort, GLP1-RA use was associated with a decrease in ALT by 69% and A1c by 25.8% over an average of 11 months. This is a notable reduction for both biomarkers, highlighting the potential for GLP-1 RAs in the management of pediatric MASLD. Current guidelines for the treatment of MASLD recommend lifestyle changes such as improvement in diet and exercise as first-line therapy [1], but, to date, there has not been any pharmacotherapeutic agent approved by a regulatory agency for the treatment of pediatric MASLD.

The pathophysiology of MASLD in children involves a cycle of increased de novo lipogenesis (DNL) and intrahepatic triglyceride content with increased plasma glucose and insulin resistance [18]. Growing evidence suggests that it is the excess insulin driving the excess DNL [19]. A striking feature in this case series is that there was a significant change in ALT and A1c with minimal change in BMI. The effect of GLP-1 RAs in improving hepatic and whole-body insulin resistance may be a key driving factor in the resolution of MASLD rather than weight loss itself. Schwimmer et al. also described improvement in hepatic steatosis and ALT independent of change in BMI in adolescent boys following an eight-week trial of a diet low in free sugar content [20]. Although the patients in this case series were on doses of a GLP-1 RA approved for treating T2DM, which are lower than the doses recommended for treating obesity, the doses used may still improve insulin sensitivity [21]. The findings above suggest the possibility of targeting improved insulin sensitivity in conjunction with weight loss to treat MASLD.

Furthermore, MASLD prevalence varies by ethnicity, with a 4-fold increase in risk for steatosis in Hispanic adolescents [3]. Prevalence also varies by sex, with increased prevalence noted in boys compared to girls [6]. In our study, six out of nine patients were Hispanic/Latino, and there was female predominance (six out of nine). GLP-1 RAs could be a potential therapeutic option for this high-risk population.

The effects of GLP-1 RAs in hepatic steatosis were tested in a randomized trial in the United Kingdom [12]. In the trial, 14 adult patients with stable T2DM and biopsy-proven MASH (metabolic-dysfunction-associated steatohepatitis) received 1.8 mg liraglutide once daily via subcutaneous injection or placebo for 12 weeks, with significant improvement in liver biochemistry, including ALT, and markers of inflammation when compared to the placebo group [12]. Liraglutide was the first GLP-1 RA approved for use of the treatment of pediatric T2DM by the FDA in 2019 [22]. Since then, several other GLP-1 RAs have been approved for pediatric use, including dulaglutide and exenatide [23]. Liraglutide and semaglutide are additionally approved for use as adjunctive therapy for weight loss in the pediatric population. A 2020 meta-analysis examining the use of GLP-1 RAs in the treatment of pediatric obesity and/or T2DM found mild gastrointestinal symptoms to be the most common side effects and no episodes of severe hypoglycemia [24]. The STEP TEEN trial also reported gastrointestinal side effects to be the most frequent [25]. Although our study objective was not to report on GLP-1 safety, we found these findings to be in line with the results in our study, with patients reporting mild gastrointestinal symptoms such as nausea or abdominal pain as the most common side effects.

Future studies for pediatric MASLD may include the use of the GLP-1 RA semaglutide. The STEP TEEN trial used semaglutide at a dose of 2.4 mg once weekly via subcutaneous injection for the treatment of obesity in adolescents. Comparatively, we had similar findings in that there was reduction in ALT with use of GLP-1 RAs [25]. However, the baseline ALT was lower in the STEP TEEN trial compared to our study, and we did not find large changes in weight. This may also be because semaglutide tends to have greater reductions in weight compared to the other GLP-1 RAs used in our study [25]. Although several patients in our practice were initiated on semaglutide, they were excluded from this study because of a lack of follow-up ALT after starting therapy or documentation of inconsistent use.

Additionally, more data are needed to evaluate how GLP-1 RAs affect steatosis and fibrosis in the pediatric population. Hachula et al. examined adults with T2DM, cardiovascular disease, and hepatic steatosis who were started on treatment with GLP-1 RAs, including semaglutide and dulaglutide [26]. They included the use of the FIB-4 score, which measures liver fibrosis, and anthropometric measurements, finding positive correlations between decreases in FIB-4 value with reduction in BMI, waist circumference, and waist–hip ratio [26]. Future studies in the pediatric population can similarly incorporate validated liver fibrosis scores, imaging like MRI and ultrasound, as well as biomarkers for MASLD.

The limitations of this case series include its small sample size, the presence of confounding variables (such as the initiation of other T2DM medications near the start of the GLP-1 RA treatment), and switching or discontinuation of the medication in follow-up visits. There were inconsistent variables due to the nature of retrospective chart reviews, including the need for a consistent number of data points for all patients, variations in time intervals between measurements for each patient, and the length of time that the patients were continued on the medication. Compliance was also determined via chart review, which may provide a partial picture.

This case series shows preliminary findings of the potential use of GLP-1 RAs for the treatment of pediatric MASLD. More randomized, placebo-controlled studies and reports with larger sample sizes are needed to better understand the effects of GLP-1 RAs on MASLD in the pediatric population.

## Data Availability

The data presented in this study are available on request from the corresponding author. The data are not publicly available due to the risk of loss of confidentiality.

## References

[1] Vos M.B., Abrams S.H., Barlow S.E., Caprio S., Daniels S.R., Kohli R., Mouzaki M., Sathya P., Schwimmer J.B., Sundaram S.S. (2017). NASPGHAN Clinical Practice Guideline for the Diagnosis and Treatment of Nonalcoholic Fatty Liver Disease in Children: Recommendations from the Expert Committee on NAFLD (ECON) and the North American Society of Pediatric Gastroenterology, Hepatology and Nutrition (NASPGHAN). J. Pediatr. Gastroenterol. Nutr..

[2] Lazarus J.V., Newsome P.N., Francque S.M., Kanwal F., Terrault N.A., Rinella M.E. (2023). A multi-society Delphi consensus statement on new fatty liver disease nomenclature. Hepatology.

[3] Wong R.J., Aguilar M., Cheung R., Perumpail R.B., Harrison S.A., Younossi Z.M., Ahmed A. (2015). Nonalcoholic steatohepatitis is the second leading etiology of liver disease among adults awaiting liver transplantation in the United States. Gastroenterology.

[4] Ciardullo S., Monti T., Perseghin G. (2021). Prevalence of Liver Steatosis and Fibrosis Detected by Transient Elastography in Adolescents in the 2017–2018 National Health and Nutrition Examination Survey. Clin. Gastroenterol. Hepatol..

[5] Ciardullo S., Carbone M., Invernizzi P., Perseghin G. (2022). Impact of the new definition of metabolic dysfunction-associated fatty liver disease on detection of significant liver fibrosis in US adolescents. Hepatol. Commun..

[6] Schwimmer J.B., Pardee P.E., Lavine J.E., Blumkin A.K., Cook S. (2008). Cardiovascular risk factors and the metabolic syndrome in pediatric nonalcoholic fatty liver disease. Circulation.

[7] Newton K.P., Hou J., Crimmins N.A., Lavine J.E., Barlow S.E., Xanthakos S.A., Africa J., Behling C., Donithan M., Clark J.M. (2016). Prevalence of Prediabetes and Type 2 Diabetes in Children With Nonalcoholic Fatty Liver Disease. JAMA Pediatr..

[8] Eslam M., Valenti L., Romeo S. (2018). Genetics and epigenetics of NAFLD and NASH: Clinical impact. J. Hepatol..

[9] Fang Y.L., Chen H., Wang C.L., Liang L. (2018). Pathogenesis of non-alcoholic fatty liver disease in children and adolescence: From “two hit theory” to “multiple hit model”. World J. Gastroenterol..

[10] Lomonaco R., Bril F., Portillo-Sanchez P., Ortiz-Lopez C., Orsak B., Biernacki D., Lo M., Suman A., Weber M.H., Cusi K. (2016). Metabolic Impact of Nonalcoholic Steatohepatitis in Obese Patients With Type 2 Diabetes. Diabetes Care.

[11] Donath M.Y., Burcelin R. (2013). GLP-1 effects on islets: Hormonal, neuronal, or paracrine?. Diabetes Care.

[12] Armstrong M.J., Hull D., Guo K., Barton D., Hazlehurst J.M., Gathercole L.L., Nasiri M., Yu J., Gough S.C., Newsome P.N. (2016). Glucagon-like peptide 1 decreases lipotoxicity in non-alcoholic steatohepatitis. J. Hepatol..

[13] Blundell J., Finlayson G., Axelsen M., Flint A., Gibbons C., Kvist T., Hjerpsted J.B. (2017). Effects of once-weekly semaglutide on appetite, energy intake, control of eating, food preference and body weight in subjects with obesity. Diabetes Obes. Metab..

[14] Aroda V.R., Ahmann A., Cariou B., Chow F., Davies M.J., Jodar E., Mehta R., Wooh V., Lingvay I. (2019). Comparative efficacy, safety, and cardiovascular outcomes with once-weekly subcutaneous semaglutide in the treatment of type 2 diabetes: Insights from the SUSTAIN 1–7 trials. Diabetes Metab..

[15] Marso S.P., Bain S.C., Consoli A., Eliaschewitz F.G., Jódar E., Leiter L.A., Lingvay I., Rosenstock J., Seufert J., Warren M.L. (2016). Semaglutide and Cardiovascular Outcomes in Patients with Type 2 Diabetes. N. Engl. J. Med..

[16] Newsome P.N., Buchholtz K., Cusi K., Linder M., Okanoue T., Ratziu V., Sanyal A.J., Sejling A.-S., Harrison S.A. (2021). A Placebo-Controlled Trial of Subcutaneous Semaglutide in Nonalcoholic Steatohepatitis. N. Engl. J. Med..

[17] Schwimmer J.B., Newton K.P., Awai H.I., Choi L.J., Garcia M.A., Ellis L.L., Vanderwall K., Fontanesi J. (2013). Paediatric gastroenterology evaluation of overweight and obese children referred from primary care for suspected non-alcoholic fatty liver disease. Aliment. Pharmacol. Ther..

[18] Smith G.I., Shankaran M., Yoshino M., Schweitzer G.G., Chondronikola M., Beals J.W., Okunade A.L., Patterson B.W., Nyangau E., Field T. (2020). Insulin resistance drives hepatic de novo lipogenesis in nonalcoholic fatty liver disease. J. Clin. Investig..

[19] Huneault H.E., Ramirez Tovar A., Sanchez-Torres C., Welsh J.A., Vos M.B. (2023). The Impact and Burden of Dietary Sugars on the Liver. Hepatol. Commun..

[20] Schwimmer J.B., Ugalde-Nicalo P., Welsh J.A., Angeles J.E., Cordero M., Harlow K.E., Alazraki A., Durelle J., Knight-Scott J., Newton K.P. (2019). Effect of a Low Free Sugar Diet vs Usual Diet on Nonalcoholic Fatty Liver Disease in Adolescent Boys: A Randomized Clinical Trial. JAMA.

[21] Bednarz K., Kowalczyk K., Cwynar M., Czapla D., Czarkowski W., Kmita D., Nowak A., Madej P. (2022). The Role of Glp-1 Receptor Agonists in Insulin Resistance with Concomitant Obesity Treatment in Polycystic Ovary Syndrome. Int. J. Mol. Sci..

[22] Bacha F. (2019). FDA approval of GLP-1 receptor agonist (liraglutide) for use in children. Lancet Child. Adolesc. Health..

[23] Berman C., Vidmar A.P., Chao L.C. (2023). Glucagon-like Peptide-1 Receptor Agonists for the Treatment of Type 2 Diabetes in Youth. Eur. Endocrinol..

[24] Chadda K.R., Cheng T.S., Ong K.K. (2021). GLP-1 agonists for obesity and type 2 diabetes in children: Systematic review and meta-analysis. Obes. Rev..

[25] Weghuber D., Barrett T., Barrientos-Pérez M., Gies I., Hesse D., Jeppesen O.K., Kelly A.S., Mastrandrea L.D., Sørrig R., Arslanian S. (2022). Once-Weekly Semaglutide in Adolescents with Obesity. N. Engl. J. Med..

[26] Hachula M., Kosowski M., Basiak M., Okopien B. (2023). Does Therapy with Glucagon-like Peptide 1 Receptor Agonists Have an Effect on Biochemical Markers of Metabolic-Dysfunction-Associated Steatotic Liver Disease (MASLD)? Pleiotropic Metabolic Effect of Novel Antidiabetic Drugs in Patients with Diabetes-Interventional Study. Pharmaceuticals.

